# Bilateral pediatric pial arteriovenous fistulas accompanying a giant arachnoid cyst with torticollis: A case report

**DOI:** 10.1097/MD.0000000000020991

**Published:** 2020-06-26

**Authors:** Junrao Li, Ting Wang, Seidu A. Richard, Changwei Zhang, Xiaodong Xie, Chaohua Wang

**Affiliations:** a Department of Neurosurgery, West China Hospital, Sichuan University, Chengdu, China; b Department of Medicine, Princefield University, Ho-Volta Region, Ghana West Africa.

**Keywords:** arachnoid cyst, coils, onxy, pial arteriovenous fistula, torticollis

## Abstract

**Rationale:**

Pial arteriovenous fistula (PAVF) occurs when intracranial arteries communicate directly with veins. PAVFs are very rare congenital vascular lesions that are commonly seen in infants and children. Arachnoid cysts are congenital cavitation often filled with cerebrospinal fluid. We present a very rare associated occurrence of bilateral pediatric PAVF and a giant arachnoid cyst presenting as torticollis in a child. So far, this is the first case.

**Patient concerns:**

A 6-year-old male child was referred to our facility from a local hospital due to severe torticollis.

**Diagnoses:**

An enhanced computed tomography scan revealed 2 slightly high-density masses at the anterior pontine cistern, right circumferential cistern, as well as left posterior occipital region. The same computed tomography scan revealed a giant arachnoid cyst in the left occipital as well as the temporal region with a thin cerebral cortex adjacent to the cyst.

**Interventions:**

Digital subtraction angiography confirmed that the 2 high-flow lesions were PAVFs. The patient was treated with a combination of detachable coils and Onyx Liquid Embolic System (Onyx HD-500) (Covidien/ev3 Neurovascular) via the transarterial endovascular route while the giant arachnoid cyst was managed conservatively.

**Outcomes:**

The torticollis resolved 2 days after the procedure. He is currently well with no neurologic deficit.

**Lessons:**

We advocate that in cases of PAVF with accompanying cyst, the cyst should be managed conservatively if it is not associated with intracranial hemorrhage or focal neurologic deficit.

## 1 Introduction

Pial arteriovenous fistula (PAVF), also known as brain arteriovenous fistula, occurs when intracranial arteries communicate directly with veins.^[1–3]^ PAVFs are very rare vascular lesions, that accounts for about 1.6% to 4.7% of all arteriovenous malformations.^[2]^ These lesions are congenital and are commonly seen in infants and children.^[3]^ The lesions often comprise of one or more direct supplying arteries, one draining vein and no abnormal vascular mass.^[4]^ In PAVF, the feeding arteries are mainly derived from the pial and cortical arteries and flow directly into a single vein to initiate high flow hemodynamic changes resulting a dilated and tortuous vein.^[4,5]^

Cerebral angiography is the gold-standard radiologic modality for the diagnosis of PAVF.^[4]^ It can determine the direction of supplying artery, drainage vein, venous drainage, fistula size, as well as possible dangerous vascular anastomosis.^[5,6]^ It can also measure blood flow velocity and pressure.^[4]^ Even in asymptomatic cases, treatment recommended because of the potentially fatal bleeding risk of the lesions.^[2]^ The most common treatment modalities include endovascular embolization, microsurgery, and radiotherapy.^[3,4]^ The goal of treatment is to occlude the fistula site or occlusion of the feeding artery and proximal drainage vein closer to the fistula.^[3,6]^

Arachnoid cysts are congenital cavitation often filled with cerebrospinal fluid (CSF).^[7,8]^ They constitute about 1% of all intracranial cystic space-occupying lesions and are normally detected incidental on radiologic imaging.^[7,8]^ In children, their prevalence rate is about 2.6% and usually causes mass effect when they advance into large or giant cysts.^[8,9]^ Smaller arachnoid cysts are managed conservatively unless, they cause compressive symptoms or brain damage while large cysts are often surgical resected.^[8,10]^ We present a very rare concomitant occurrence of bilateral pediatric PAVF and a giant arachnoid cyst with torticollis.

## 2 Case report

A 6-year-old male child was referred to our facility from a local hospital due to severe torticollis. Prior to this, the patient had no history of head trauma or craniocerebral infection. No obvious neurologic defects were found during physical examination. Detailed cranial nerve as well as ophthalmic examination did not yield much. No murmurs were heard on auscultation, and the family denied family history of any hereditary disease. Laboratory investigations were at normal ranges. Chest X-ray and electrocardiogram were normal.

**Figure 1:**
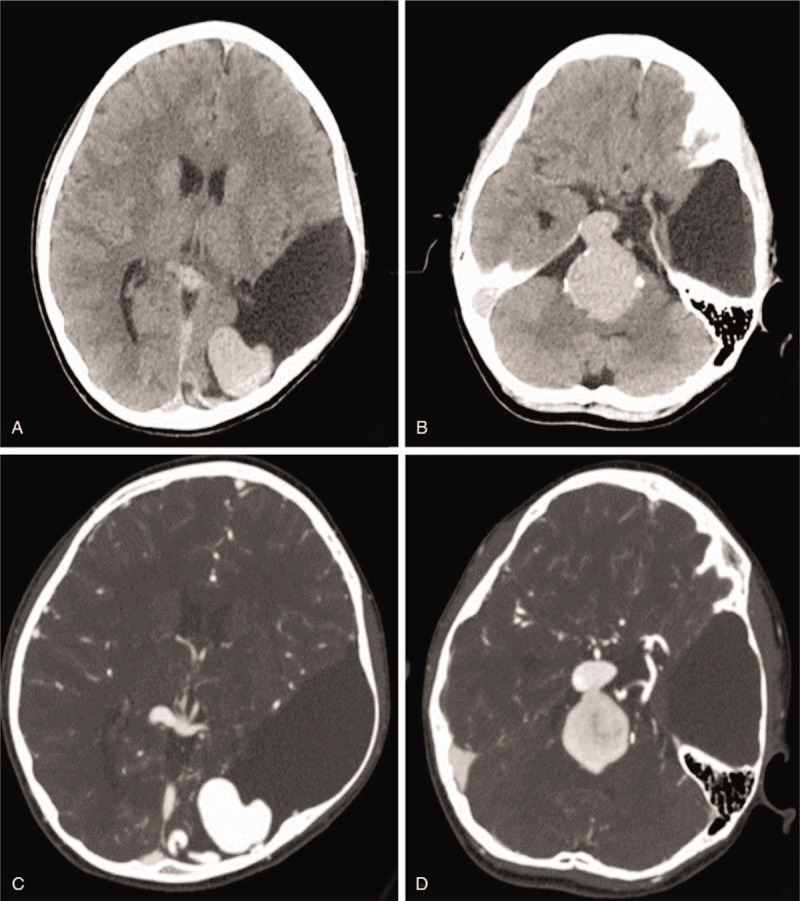
Computed tomography (CT) scan images showing the bilateral pial arteriovenous fistulas accompanying a giant arachnoid cyst in the left occipital and temporal region. (A, B) Two slightly high-density masses at the anterior pontine cistern, right circumferential cistern, and left posterior occipital region. (C, D) Enhanced CT scan showing the degree of vascular enhancement.

**Figure 2:**
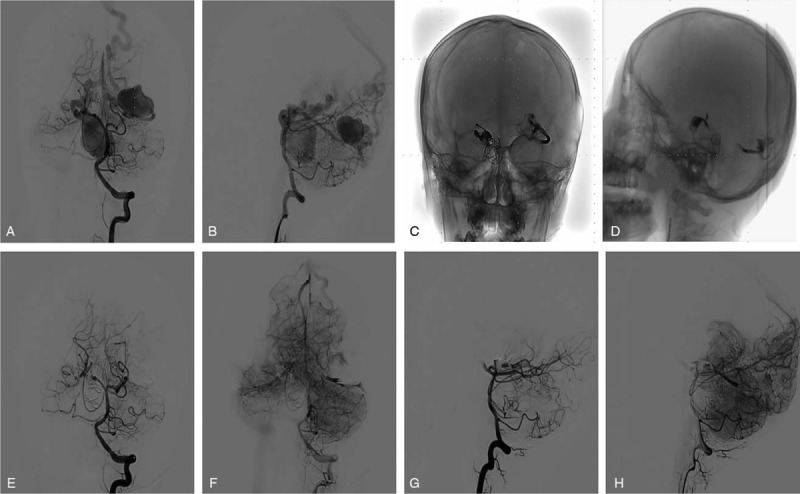
Diagnostic, operative, as well as postoperative digital subtraction angiography (DSA) images of the pial arteriovenous fistulas (PAVFs). (A, B) DSA showing the 2 high-flow PAVFs. (C, D) Operative DSA image showing coils and Onyx agent used to occlude the both PAVFs. (E–H) Postoperative angiography showing total occlusion of both fistulas.

An enhanced computed tomography (CT) scan revealed 2 slightly high-density masses at the anterior pontine cistern, right circumferential cistern, as well as left posterior occipital region (Fig. 1 A, B). These masses where consistent with the degree of vascular enhancement so we mistook the lesions for vascular malformations (Fig. 1C, D). The same CT scan revealed a giant arachnoid cyst in the left occipital as well as the temporal region with a thin cerebral cortex adjacent to the cyst (Fig. 1A–D). Digital subtraction angiography (DSA) also revealed 2 high-flow PAVFs (Fig. 2 A, B). The first one was located on the anterior slope of the brainstem with blood supply from the right posterior cerebral artery, and drained into the straight sinus via the peri-pons vein. The other was located on the left occipital lobe with blood supply from the left posterior cerebral artery and drained into the superior sagittal sinus via the occipital cortical vein.

The patient was treated with a combination of detachable coils and Onyx Liquid Embolic System (Onyx HD-500) (Covidien/ev3 Neurovascular) via the transarterial endovascular route. He was put on aspirin and clopidogrel per kilogram body daily for at least 5 days prior to the procedures. The entire procedure was done under general anesthesia with constant heparin infusion. After securing an arterial line on the right femoral artery a 5-French femoral arterial sheath was placed and coiling as well as embolization was performed via selective catheterization through the right posterior cerebral artery (Fig. 2 C, D). Coils were released very close to the fistula site followed by diffused Onyx Liquid Embolic System at the right posterior cerebral artery into the fistula. The whole procedure was repeated for the fistula at left occipital lobe via the left posterior cerebral artery.

**Figure 3:**
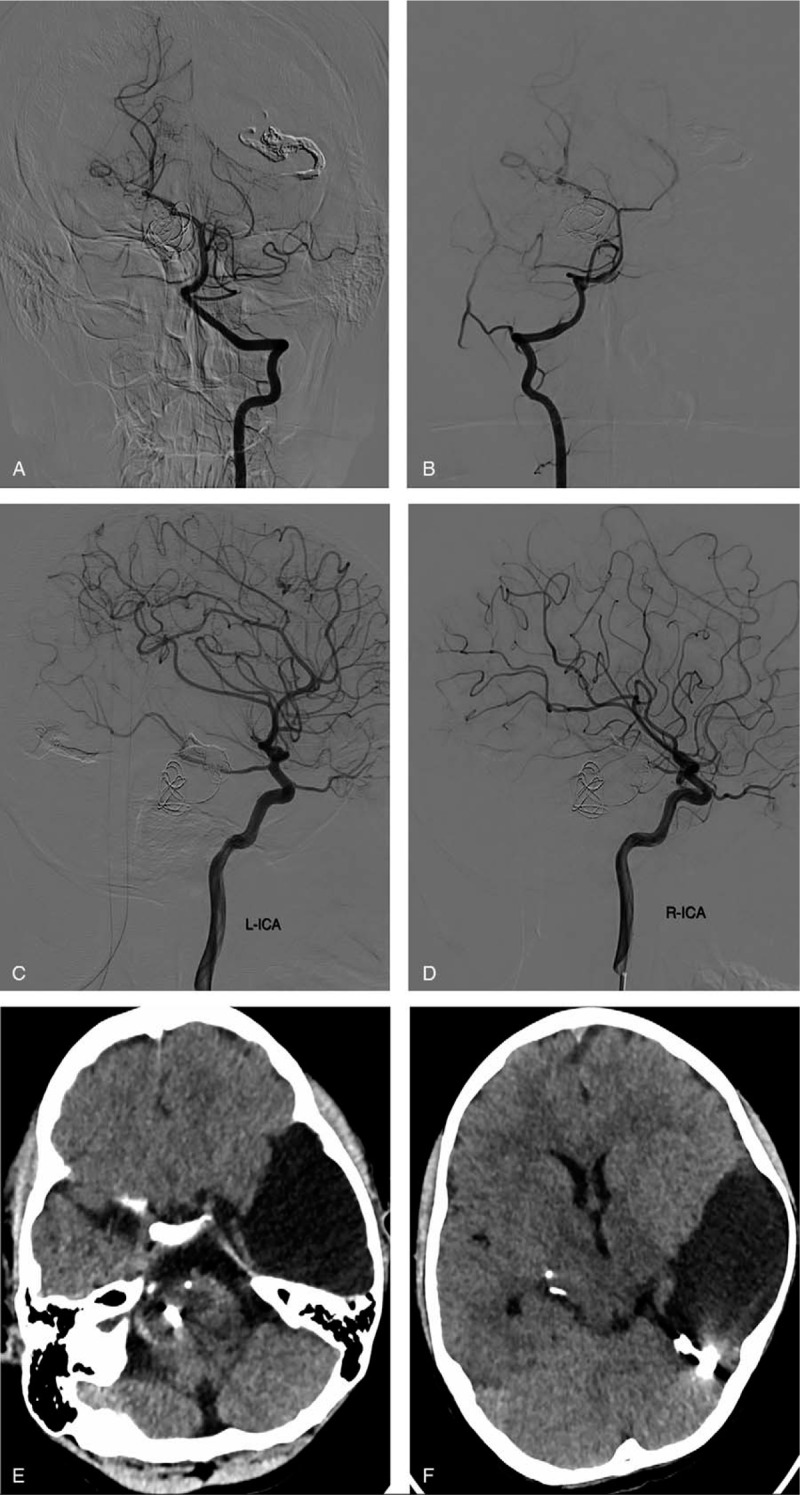
Six months follow-up images. (A–D) digital subtraction angiography images showing total disappearance of the biteral pial arteriovenous fistulas. (E, F) Computed tomography scan images showing no regression of the cyst.

Nevertheless, the accompanying giant cyst was managed conservatively because it not presents with any associated intracranial hemorrhage or any focal neurologic deficit. Immediate postoperative angiography revealed total occlusion of both fistulas (Fig. 2E–H). Two days after the procedure, we observe a resolution of the torticollis. He was discharged home on the first day after the procedure with no further neurologic deficit. Six months follow-up angiograph show total disappearance of the bilateral PAVF (Fig. 3A–D) while CT scan images showed no regression of the cyst (Fig. 3 E, F). He is currently well with no neurologic deficit.

## 3 Discussion

Initially, PAVF was referred to as fistulous arteriovenous malformation (AVM) and classified as a type of AVM.^[4]^ Current investigations have shown that PAVFs have arterial feeders, nidus, as well as drainage veins and thus are not “true” AVMs.^[4]^ Varix formation is a unique finding in almost all patients with PAVF.^[4]^ Several authors have associated the formation of varix to high-pressure blood flow from arterial feeder straight into the venous drainage.^[4,6,11]^

On the contrary, arachnoid cysts are benign, extra axial, cystic disorders which occur as a result of congenital separation of the arachnoid layer.^[10,12]^ They are categorized into primary and secondary cysts. The primary type occurs as a result of separation of the arachnoid membranes in-utero leading to cystic cavitation and subsequent anomalous collection of CSFs. The secondary type occurs subsequent to surgery, trauma, infection, or intracranial hemorrhage.^[10,13]^ The separation of arachnoid membranes occurs when mesoectodermal tissue detaches during the folding of the neural tube.^[12]^

Congenital hereditary vascular disorders have been observed in patients with PAVFs.^[4]^ Also, congenital anomalies involving the corpus callosum, callosal agenesis, as well as Chiari malformations with neurofibromatosis type 1 have associated with arachnoid cysts.^[10,12]^ This is first case of concomitant occurrence of a pediatric PAVF and a giant arachnoid cyst. The clinical presentation of PAVF differs according to patient's age as well as the existence of a varix.^[2,4,14]^ Infancy often present with focal neurologic deficits and increased head circumference while children and adults present with headaches, space occupying effect, seizures, cerebral hemorrhage, as well as focal neurologic deficits.^[2,5]^

The clinical presentation of arachnoid cysts differs according to their size as well as location.^[10]^ Small cysts are usually symptomatic while large or giant cyst causes mass effect on neurovascular structures. Huge cyst may also impede the function of adjacent brain. It may rupture resulting in intracystic hemorrhage or subdural hemorrhage resulting in abrupt life-threatening neurologic deficits.^[10,13]^ Patients initially present with headaches, dizziness, nausea, vomiting, seizures, ataxia, hearing loss worsening of mood, as well as mental status changes.^[9,12]^ The most cardinal clinical presentation in our case was torticollis. This is the first case of PAVFs accompanied by giant arachnoid cyst manifesting as torticollis. The torticollis may have occurred as result of compression at the foramina magnum by the cyst.

On CT scan, PAVFs usually have homogeneous contrast enhancement with characteristic expansion of the drainage vein.^[3]^ CT scan can also able to detect associated cerebral hemorrhage, hydrocephalus, as well as encephalatrophy.^[3,15]^ Nevertheless, arachnoid cysts are seen on CT scan as extra-axial CSF dense lesions triggering local space occupying effect as well as sometimes calvarial modification.^[9,10,13]^ In our case, an enhanced CT scan was very valuable because it revealed 2 slightly high-density masses at the anterior pontine cistern, right circumferential cistern, as well as left posterior occipital region which prompted us to perform a diagnostic DSA.

Magnetic resonance imaging (MRI) is a very valuable radiologic modality for further evaluation of PAVFs or arachnoid cysts after CT had made the initial detection.^[3]^ In patients with PAVFs, MRI is able to identify the anatomic location, feeders, venous varix as well as regional, hemispheric, or diffuse cerebral malacia.^[3,16]^ Nevertheless, MRI is unable to detect flow-empty actions produced by a nidus.^[16]^ On the contrary, MRI is gold-standard radiologic modality for the evaluation of arachnoid cysts. MRI is able to confirm the extra-axial site as well as typical T2-weighted signal of the cyst analogous to that of CSF.^[10]^ We did not further assess the patient with MRI because our facility has DSA hence DSA is the gold-standard radiologic modality for assessing PAVFs.

Computed tomography angiography (CTA) is very valuable during the evaluation of PAVFs because it is able to outline the complex angioarchitecture.^[5]^ Magnetic resonance angiography is also capable of delineating clearly the feeding arteries and veins of PAVF.^[3]^ Nevertheless, both magnetic resonance angiography and CTA produce static images and are incapable of recording the hemodynamic features of PAVFs. However, time-determined contrast-enhanced magnetic resonance DSA (MRDSA) can efficiently eliminate these difficulties during the evaluation of PAVFs.^[3,17]^ DSA is therefore the gold-standard imaging modality for PAVFs. DSA is capable of delineating clearly the feeding arteries and veins of PAVF as well as the hemodynamic features at different time phases.^[3,17]^ We utilized DSA to confirm the diagnosis of the PAVFs in our case.

Surgery often involves resection of varix as well as ligation of both arterial feeder and drainage of the PAVF.^[4,6,18]^ In cases where the arterial feeder is a short branch of a cortical artery and cannot be occluded, microsurgical approaches are often advocated.^[6]^ Nevertheless, endovascular route is still the most preferred treatment option for PAVFs. Endovascular treatment option is very simple and safe.^[3]^ Endovascular treatment involves the transartery as well as the transvenous routes. In cases of multiple arterial connections or high flow feeder, it often difficult to eliminate the PAVF via the transarterial approach.^[4,6]^

The most favorably embolic agents used during endovascular therapy are detachable coils and *N*-butyl-2-cyanoacrylate.^[18]^ Nevertheless, balloons have also been used effectively to control as well as occlude the flow of feeding artery.^[2]^ The placement of detachable coils is very effective and efficient. Coils are not easily conveyed away by the high-speed blood flow associated with PAVFs.^[3,14]^ Onyx embolization is another effective and efficient embolic agent for the treatment of high-flow PAVFs.^[2,3]^ It has the capability of redirecting the flow during delivery as well as allowing for exact delivery into the fistula location.^[3]^ It is advocated that a combination of coils and *N*-butyl-2-cyanoacrylate should be used in treating selected high-velocity PAVF with complex architectures.^[3]^ In some complex case of PAVFs, a combination of surgery and endovascular treatment aided by intraoperative DSA in a hybrid theater is often the most effective treatment option.^[3]^ We successfully treated our patients with a combination of detachable coils and Onyx Liquid Embolic System via the transarterial endovascular route.

The treatment of arachnoid cysts often includes conservative as well as surgery.^[10]^ Conservative treatment is reserved for small and nonsymptomatic cysts.^[13]^ Surgical treatment often depends on the size, location, as well as the operative risk profile.

Surgery is advocated for patients with focal neurologic symptoms as well as their readiness to avoid a shunt and the chance of recurrence.^[10,12]^ The most appropriate surgical technique for arachnoid cysts is fenestration.^[19]^ In fenestration, a passage is created to allow for communication between cyst and the subarachnoid space or ventricle. This technique is most suitable for arachnoid cysts in almost every location.^[12,19]^ Nevertheless, ventriculocystostomy or ventriculo-cystocysternostomy is long-term treatments for cysts located in the suprasellar region.^[10]^ In our case, the accompanying giant cyst was managed conservatively because it does not present with associated intracranial hemorrhage or any focal neurologic deficit.

## 4 Conclusion

Concomitant occurrence of bilateral pediatric PAVF and a giant arachnoid cyst is very rare and so far, this is the first case. The bilateral PAVFs were successfully treated with a combination of detachable coils and Onyx Liquid Embolic System via the transarterial endovascular route. Nevertheless, the accompanying giant cyst was managed conservatively because it does not present with associated intracranial hemorrhage or any focal neurologic deficit.

## Author contributions

All authors contributed toward data analysis, drafting and critically revising the paper and agree to be accountable for all aspects of the work. Seidu A. Richard and Junrao Li wrote the final paper. All authors approved the final version of this paper.

**Conceptualization:** Junrao Li, Ting Wang, Seidu A. Richard, Changwei Zhang, Xiaodong Xie, Chaohua Wang.

**Data curation:** Junrao Li, Ting Wang, Seidu A. Richard, Changwei Zhang, Xiaodong Xie, Chaohua Wang.

**Formal analysis:** Junrao Li, Ting Wang, Seidu A. Richard, Changwei Zhang, Xiaodong Xie, Chaohua Wang.

**Methodology:** Seidu A. Richard.

**Resources:** Changwei Zhang, Xiaodong Xie, Chaohua Wang.

**Supervision:** Changwei Zhang, Xiaodong Xie, Chaohua Wang.

**Writing – original draft:** Seidu A. Richard.

**Writing – review & editing:** Junrao Li, Ting Wang, Seidu A. Richard, Changwei Zhang, Xiaodong Xie, Chaohua Wang.
